# Acquired Reactive Perforating Collagenosis—A Rare Entity Occurring Within Common Disorders: A Systematic Review and Our Personal Experience

**DOI:** 10.3390/jcm15010391

**Published:** 2026-01-05

**Authors:** Maria Alexandra Junghetu, Cristina Violeta Tutunaru, Simona Laura Ianoși, Claudia Valentina Georgescu, Olguța Anca Orzan

**Affiliations:** 1 Department of Oncologic Dermatology, “Carol Davila” University of Medicine and Pharmacy, 020021 Bucharest, Romania; maria-alexandra.junghetu0425@rez.umfcd.ro (M.A.J.); olguta.orzan@umfcd.ro (O.A.O.); 2 Department of Dermatology, Elias University Emergency Hospital, 011461 Bucharest, Romania; 3 Department of Dermatology, Faculty of Medicine, University of Medicine and Pharmacy of Craiova, 200349 Craiova, Romania; simonaianosi@hotmail.com; 4 Department of Pathology, University of Medicine and Pharmacy of Craiova, 200349 Craiova, Romania; cgeorgescu2001@yahoo.com

**Keywords:** acquired reactive perforating collagenosis, perforating diseases, acquired perforating dermatosis

## Abstract

**Background/Objectives**: Acquired reactive perforating collagenosis (ARPC) is a rare entity usually occurring in adults with systemic diseases such as diabetes mellitus, chronic kidney disease (CKD), cardiovascular diseases, and malignancies, although drug-related and trauma-induced cases have also been reported. Given its rarity and the lack of consensus on optimal management, we conducted a systematic review to summarize updated diagnostic and therapeutic insights into ARPC. Additionally, we report a case of ARPC associated with CKD. **Methods**: This study was conducted in accordance with the PRISMA 2020 (Preferred Reporting Items for Systematic Reviews and Meta-Analyses) guidelines. A literature search was performed in the PubMed database between May–September 2025. The search strategy targeted open-access, primary human studies, published within the last 15 years, available in English, and including adult patients with histopathologically confirmed ARPC. **Results**: Twenty-seven studies, predominantly case reports and case series, were included. The mean patient age was 60.8 ± 14.4 years. Only one case occurred in the absence of comorbidities, while most subjects had underlying systemic diseases. Drug-induced cases were also described. Clinically, ARPC should be suspected in patients presenting with pruritic papules/nodules with central keratotic plugs. Additional diagnostic tools include dermoscopy and reflectance confocal microscopy. However, histopathological evidence of transepidermal elimination of altered collagen fibers is mandatory. The current treatments of ARPC include antihistamines, keratolytics, topical/intralesional/oral corticosteroids, topical/systemic retinoids, phototherapy, dupilumab and allopurinol. Other therapies have been reported across the literature, including emerging ones. **Conclusions**: Once ARPC is diagnosed, a thorough evaluation for underlying diseases, including malignancies, is essential. Clinical trials are warranted to define optimal therapeutic strategies.

## 1. Introduction

Acquired reactive perforating collagenosis (ARPC) is a subtype of the perforating disorders, characterized by transepidermal expulsion of altered collagen bundles [1,2,3,4,5]. This entity occurs in adults, mostly middle-aged, with systemic diseases such as diabetes mellitus (DM) or end-stage renal disease (ESRD), although an association with malignancies, drugs or skin trauma has been described [1,2,3,6,7,8,9,10].

Faver proposed the following diagnostic criteria for ARPC: “(1) umbilicated papules/nodules with central, adherent keratotic plug, (2) onset > 18 years and (3) histopathologic evidence of expulsion of necrotic basophilic collagen fibers into a cup-shaped epidermal depression” (see Figure S1) [11]. The skin lesions in ARPC are almost always pruritic and have a predilection for the extensor surfaces. The lesions might exhibit the Koebner phenomenon [1,2,3].

ARPC should mainly be differentiated from the other types of acquired perforating dermatoses (APDs) such as elastosis perforans serpiginosa (EPS), perforating folliculitis and Kyrle disease [4,12,13,14,15]. Other differential diagnoses include folliculitis, arthropod bites, prurigo nodularis, multiple keratoacanthomas, lichen planus and secondary perforating disorders [2,3,4,12,13,15].

Treatment of ARPC is based on symptomatic relief and management of the underlying systemic disease. ARPC has been hypothesized to be a reactive pattern to scratching from chronic pruritus [16,17]. Disruption of the itch–excoriation cycle is essential in order to stop the perpetuation of this disease. Other types of skin trauma, including superficial infections, might cause focal necrobiosis, which leads to transepithelial elimination of collagen fibers [18,19,20]. It appears that local trauma triggers the lesions by exposing the keratinocytes to modified extracellular matrix proteins [3,21]. Other factors that predispose to focal necrobiosis are DM and hypoxic conditions. Although histologically altered, collagen bundles have been proven to be ultrastructurally intact [22].

Intradermal deposition of metabolites in chronic kidney disease (CKD) might also precipitate ARPC [2,3]. Studies have identified calcium salts, uric acid or even silicon as triggers for APDs [3,23]. Moreover, increased tissular fibronectin, as seen in uremia or in diabetes, has been proposed as another mechanism behind ARPC [2,3].

It also appears that transforming growth factor beta (TGF-β), especially the β3-isoform, is overexpressed in ARPC lesions, indicating that factors that orchestrate tissue remodeling play a crucial role in this perforating disease [13,19,24]. Moreover, up-regulation of TGF-β might result as a consequence of coexisting systemic diseases such as diabetes [13]. Furthermore, TGF-β induces the synthesis of metalloproteinase-1 (MMP-1), which consequently degrades the interstitial collagen [25,26].

## 2. Methods

### 2.1. Study Design

This study was conducted in accordance with the PRISMA 2020 (Preferred Reporting Items for Systematic Reviews and Meta-Analyses) guidelines (see Table S2). The aim was to synthesize the current diagnostic and therapeutic insights into ARPC.

### 2.2. Search Strategy

A comprehensive literature search was performed in the PubMed database between May 2025 and September 2025. The search strategy included both Medical Subject Headings (MeSH) and free-text terms related to three main topics: “reactive perforating collagenosis,” “acquired reactive perforating collagenosis,” and “acquired perforating dermatosis.” Filters were applied to restrict the results to English-language studies conducted in humans and published within the last 15 years. The exact search formula is reported below:

<<“reactive perforating collagenosis”[All Fields] OR “acquired reactive perforating collagenosis”[All Fields] OR “acquired perforating dermatos*”[All Fields]>>

### 2.3. Eligibility Criteria

Studies were included if they met all of the following criteria: (1) participants aged > 18 years; (2) presence of papules or nodules with central keratotic plugs; (3) histopathological confirmation showing collagen fibers extruding through a cup-shaped epidermal invagination; (4) in vivo research; and (5) any study design except for secondary studies (e.g., reviews, meta-analyses).

### 2.4. Data Extraction

The extracted data included study characteristics (authors, design, sample size), participant demographics (age, sex), clinical features (comorbidities, lesion localization, distribution, and associated symptoms), and therapeutic approaches.

**Figure 1:**
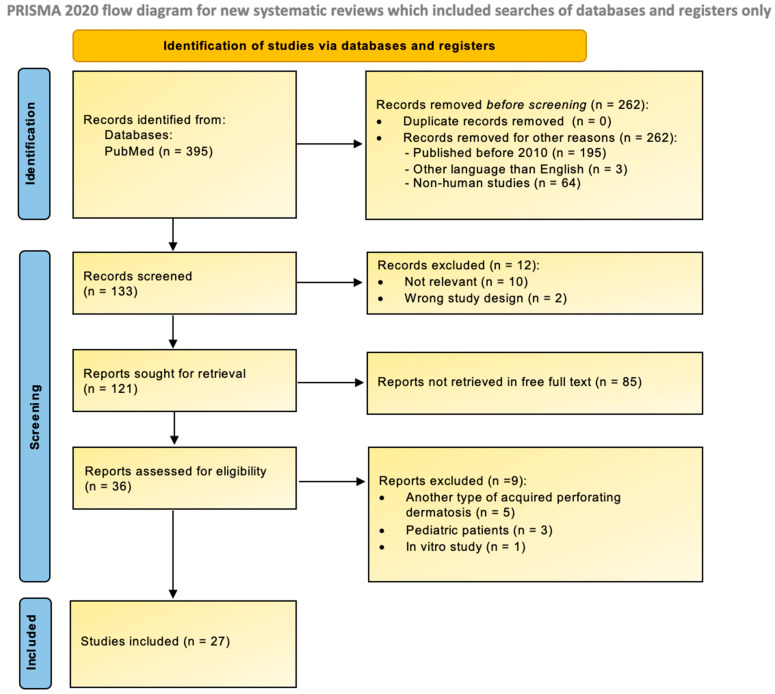
PRISMA 2020 flow diagram.

A detailed description of the methodology is provided below (Figure 1).

## 3. Results

A total of 27 studies were included in this review: one retrospective cohort study, one case-control study, two case series, 22 case reports, and one short communication. Although this systematic review provides a comprehensive overview of recent data on ARPC, it has several limitations, primarily the inclusion of low-level evidence studies such as case reports and case series. A formal risk-of-bias assessment was not conducted, as case reports inherently face a high risk of bias due to the lack of comparator groups. However, given the rarity of ARPC, the scarcity of higher-quality research is understandable. A further limitation is that certain important studies might have been inadvertently excluded due to the selection of only open-access articles. A detailed summary of the reviewed studies is presented in Table S1.

### 3.1. Findings of the Included Studies

The only included retrospective cohort study compared the improvement in the investigator global assessment (IGA) score and in the numerical rating scale (NRS) score for pruritus in patients with ARPC treated with dupilumab as monotherapy vs. conventional therapy (antihistamines and topical corticosteroids) [27]. At week 12, all the patients treated with dupilumab achieved an IGA score of 0/1, while none of the patients from the conventional therapy group achieved such a score (*p* < 0.05). Pruritus was alleviated at week 12, with at least four points in the NRS score in 90% of the subjects treated with dupilumab compared to only 10% of the patients from the second group (*p* < 0.05). Furthermore, the authors assessed by immunohistochemistry skin samples from the patients with ARPC and compared them with samples from patients with atopic dermatitis, as well as with healthy controls. They found an overexpression of type 2 inflammatory cytokines (such as IL-4 and IL-13) and Th2 cells in patients with ARPC compared to healthy controls [27].

The case control study also focused on immunohistochemistry, which had been performed on skin samples from patients with ARPC, as well as from healthy controls, to determine, semi-quantitatively, the expression of the receptor for advanced glycation end products (RAGE). RAGE was overexpressed in endothelial cells, inflammatory cells and fibroblasts of the dermis of the subjects with ARPC compared to healthy controls (*p* < 0.05) [28].

One of the included case series reported 37 patients with APDs [29]. The lesions were most frequently found on the lower limbs, followed by the upper limbs and the trunk. The vast majority of the subjects presented with pruritus. Moreover, 86% of the patients had a coexisting systemic disease. DM had the highest prevalence, followed by thyroid nodules, hypertension, allergic dermatoses, CKD and malignancies such as rectal cancer, prostate cancer or thyroid cancer. Additionally, 73% of the cases with APDs presented with transepidermal extrusion of only collagen fibers, classifying these cases as ARPC. Dermoscopy revealed different patterns based on the stage of the lesions: developing, recovery and healing lesions. In fully developed lesions of APDs, the authors observed central ulcerations/crusts surrounded by loop (hairpin) vessels arranged in a garland pattern or, less frequently, ulcerated lesions surrounded by branched or dotted vessels. In the recovery phase, there was a reduction in the peripheral vascularity, with the lesions being surrounded by dark red unstructured rings/patches. In healed lesions, the central crust fell off, leaving an atrophic scar surrounded by peripheral hyperpigmentation. Reflectance confocal microscopy (RCM) had shown hyperreflective cord-like structures from dermis to epidermis in four subjects, which disappeared after treatment. The therapeutic management included (1) topical corticosteroids along with antihistamines and NB-UVB, (2) oral tretinoin or (3) dupilumab [29].

The other case series also reported patients with APDs [30]. ARPC was the most common type (48%) of APD found in this cohort. The lower limbs were the most frequently implicated site, followed by the upper limbs and the trunk. A significant association between the affected area and the type of APD was found: 87% of the patients with ARPC had lower limb involvement (*p* < 0.05). Intense pruritus characterized the majority of the cohort, while most of the patients with itchy lesions had the ARPC subtype. Almost all the subjects had a coexisting systemic disease (90%). Most patients with associated hypertension, chronic venous insufficiency or DM had ARPC. Patients with ARPC had poorer responses when treated with allopurinol compared to those with EPS. Although therapeutic management of comorbidities, discontinuation of any incriminated drug, topical therapy (emollients ± topical corticosteroids ± keratolytic agents) and, eventually, oral antihistamines are the first-line therapy for any type of APD, a different treatment algorithm was proposed for the second- and the third-line therapy based on the subtype of APD. For ARPC, topical therapy (tretinoin or intralesional steroid injection) was advised in case of first-step failure. For non-responders to topical treatment, the authors suggested oral corticosteroids, acitretin or phototherapy (PUVA, NB-UVB, PDT) [30].

While the majority of the included case reports presented patients with ARPC in association with other underlying diseases, Gontijo JRV et al. reported a case of ARPC following a minor trauma (hair removal) [31].

Another insult-related case of ARPC was reported by Ghorpade AK [18]. The author described an isotopic response of Wolf at the site of some healed lesions of herpes zoster. Clearance of the ARPC lesions was achieved by 3 months of therapy with topical retinoids [18].

ARPC following an infection was also mentioned by Ye B et al. [32]. Although no fungus was identified when ARPC debuted, the patient had a recent history of locally treated tinea pedis of his left foot. Therefore, oral itraconazole was started and the ulcerated lesions rapidly responded [32,33].

Cases of ARPC in conjunction with poorly controlled DM have been reported by several authors [34,35,36,37,38,39]. Ambalathinkal JJ et al. reported a diabetic patient presenting with ARPC strictly localized on an unusual site: the lower back [34]. Fei C et al. presented a case of ARPC associated with DM, which had responded to topical steroids, oral antihistamines and compound glycyrrhizin tablets [35]. McClure SP et al. reported a diabetic patient presenting with ARPC lesions on her right lower leg. Multiple rounds of clobetasol ointment led to stabilization of the lesions [39]. Another case of unilateral ARPC lesions in a diabetic patient was described by Zhang LW et al. [37]. What is peculiar about this case is the fact that ARPC had been the only clinical manifestation that led to the diagnosis of DM [37]. Hasbún C et al. screened for associated neoplasms in their diabetic patient with ARPC, given the present lymphadenopathies [36]. Management of the DM, as well as therapy with antihistamines and triamcinolone, was performed [36].

ARPC developing in diabetic patients with CKD has been reported as well. Zhang X et al. initially treated ARPC with dermatocorticoids and oral antihistamines and by treating the diabetes, the ESRD and the associated hypothyroidism [38]. Partial response of the pruritus with no response of the lesions required the supplementation of the therapy with topical retinoic acid, zinc oxide ointment and Qingpeng ointment, with consequent alleviation [38].

Tilz H et al. described another therapeutic regimen in a patient with chronic renal failure who developed ARPC on the whole body [40]. After the failure of oral antihistamines, topical steroids and NB-UVB phototherapy, treatment with oral allopurinol was started, with the clinical response being maintained at month 14 of therapy [40]. By contrast, Kreuter A et al. obtained almost 100% skin clearance of ARPC in a patient with ESRD by debriding the necrotic debris and by using dermatocorticoids in combination with NB-UVB sessions [15].

An association between ARPC and atopic dermatitis/other eczema was highlighted in several case reports. Gil-Lianes J et al. described a patient with ARPC with a history of atopic dermatitis in childhood [41]. Eosinophilia and elevated serum IgE were present in this otherwise healthy patient. After failing to respond to topical and oral corticosteroids, NB-UVB, PUVA, antihistamines or cyclosporine, subcutaneous treatment with dupilumab was initiated, along with 6 weeks of NB-UVB, with a significant response [41]. Furthermore, Ying Y et al. reported two patients with senile atopic dermatitis who developed ARPC [42]. While both of them had associated type 2 DM, one patient had also been suffering from cardiovascular diseases. Both patients failed to respond to conventional therapies (oral antihistamines, dermatocorticoids, NB-UVB), but achieved relief after initiating dupilumab [42]. Another successfully treated case of ARPC coexisting with eczema, as well as with DM and coronary artery disease, was reported by Zheng J et al. [43]. A complete resolution of the lesions was achieved by the 8th week of treatment with oral baricitinib [43].

ARPC has been described in paraneoplastic conditions, as well as in oncologic patients, especially in association with several antineoplastic agents. Kikuchi N et al. reported two cases of ARPC in patients with clinically amyopathic dermatomyositis [44]. One case had positive antibodies anti-hepatitis C virus (HCV), while the second case had a known history of interstitial lung disease. However, screening for internal malignancies was negative in both of them [44]. Huseynova L et al. reported ARPC in a male with chronic lymphocytic leukemia, prostate adenocarcinoma and Graves’ disease [45]. The patient denied taking any medications and responded excellently to oral gabapentin and topical doxepin [45]. On the contrary, incriminated drugs have been found in several case reports of ARPC. Jiang X et al. reported a patient with lung adenocarcinoma who developed both an acneiform eruption and ARPC lesions after she had started treatment with oral erlotinib [46]. ARPC responded optimally to oral isotretinoin, topical isotretinoin and topical mometasone. After discontinuing erlotinib following cancer recovery, no recurrence of ARPC was recorded [46]. Another study reported erlotinib-induced ARPC in a patient with metastatic small-cell lung carcinoma, well-controlled type 2 DM and vitiligo [47]. Sorafenib was involved in developing ARPC in a patient with hepatocellular carcinoma associated well-controlled HIV infection and chronic HCV infection [21]. Moreover, Vega Díez D et al. reported another case of sorafenib-induced ARPC in a patient with stage IV hepatocellular carcinoma [48]. Resolution of ARPC was obtained with oral antihistamines and dermatocorticoids without the cessation of sorafenib [48].

Several case reports also focused on the additional diagnostic tools for ARPC, such as dermoscopy. Su Y et al. reported a case of ARPC associated with hypertension, ischemic heart disease and DM [49]. Dermoscopy revealed a central yellow–brown bleeding crust, surrounded by a whitish edge or a collar-like scale and, more peripherally, a pink structureless area with dotted vessels. Therapy consisted of oral corticosteroids, oral antihistamines, oral pregabalin and topical corticosteroids [49]. Furthermore, Wang C et al. reported a patient with ARPC, alcoholism and Meniere’s disease [14]. Dermoscopic examination showed a red–brown structureless area (covered by hemorrhagic crusts) with a white rim peripherally, all surrounded by an erythematous circle with present vascularization (looped and dotted vessels). Clearance of the lesions was obtained following 5-week therapy with oral antihistamines, dermatocorticoids and NB-UVB phototherapy. Unfortunately, the ARPC relapsed after the patient resumed alcohol consumption [14].

In a short communication paper, the authors stated the possibility of developing ARPC as a result of long-term therapy with superpotent dermatocorticoids for skin conditions such as eczema or prurigo nodularis [50]. They had observed such a correlation in their patients over the years, as well as a higher incidence of ARPC when using corticosteroid ointments compared to creams, probably due to the stronger effects as a result of the occlusive properties of such formulations. Madanchi M. et al. suggested that the connection between ARPC and very strong potency is based on a disrupted microcirculation as a result of the intense vasoconstriction induced by such corticosteroids. They also proposed switching from very strong dermatocorticoids to stronger/milder ones after developing ARPC [50].

### 3.2. Our Personal Experience with ARPC—A Case Report

**Figure 2:**
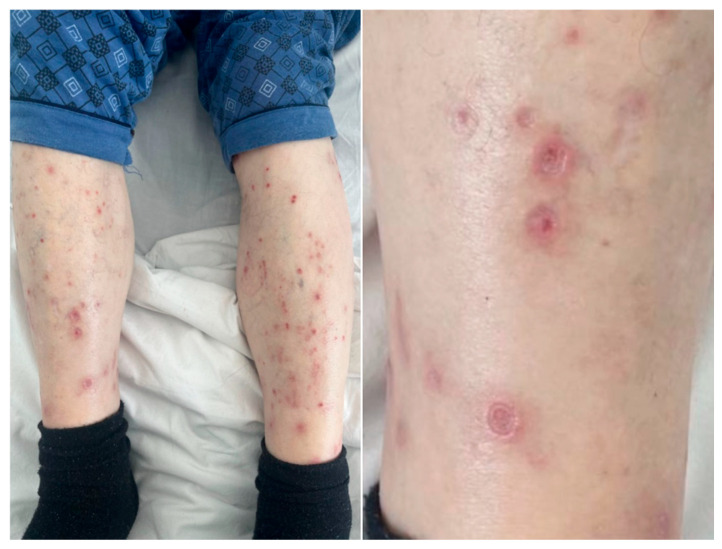
Erythematous–violaceous papules and nodules with central ulceration and hemorrhagic crusts, associated with atrophic, hypopigmented scars.

An 84-year-old male patient with no medical history or previous hospitalizations presented with an intensely pruritic polymorphous rash consisting of erythematous papules and plaques with erosions, scales and crusts on the surface, disseminated on the trunk and on the upper limbs. Following a complete body examination, we found a relatively symmetrical rash disseminated on the lower limbs, consisting of erythematous–violaceous papules and nodules, some with central ulceration on the surface, others covered by hemorrhagic crusts, accompanied by several atrophic, hypopigmented scars (Figure 2). The itchy lesions first appeared 2 months prior to this hospital admission. The patient denied the use of topical therapies for this rash, as well as the use of any systemic drugs. No obvious trigger was identified, considering the fact that the patient denied any history of infections prior to the onset of the rash, any medication or any environmental factor with which he had come into contact over the last 2 months.

Laboratory tests revealed increased serum creatinine (1.91 mg/dL), increased blood urea nitrogen (117.70 mg/dL), hyperuricemia (11.1 mg/dL) and mild anemia. We also performed a biopsy from an ulcerated lesion on the left leg. Histopathological examination revealed a skin fragment with an area of epidermal invagination covered by polymorphonuclear cells, necrotic debris, rare erythrocytes and a few eosinophilic fibers cut transversely. Acanthosis and pseudoepitheliomatous hyperplasia of the epidermis were observed. Additionally, rare vertically oriented eosinophilic fibers surrounded by a granulomatous foreign-body-type inflammatory reaction were noted. In the mid and deep dermis, thick bands of collagen sclerosis and a perivascular and periadnexal inflammatory infiltrate were evident, along with ectatic capillaries, suggesting a reactive perforating collagenosis. Masson’s trichrome staining was performed for a definitive diagnosis.

Based on the clinical appearance of the lesions on the lower limbs and considering the newly diagnosed chronic kidney disease, we oriented our diagnosis toward a perforating dermatosis. Subsequently, pathology confirmed the diagnosis of ARPC.

Following a nephrology consultation, treatment with oral allopurinol (300 mg once a day) and acetylcysteine (300 mg/day) was started. Systemic therapy with corticosteroids (dexamethasone for 8 days), gastroprotective agents and oral antihistamines was initiated. Topical therapy included clobetasol and emollients. During hospitalization, the course of the lesions and the pruritus was favorable. Subsequently, the patient was discharged with the following treatment plan: topical therapy with dermatocorticoids (hydrocortisone butyrate cream) and emollients and systemic therapy with oral antihistamines (bilastine 20 mg in the morning + ketotifen 1 mg in the evening) and oral allopurinol (300 mg/day). The patient was scheduled for follow-up, but he did not present for evaluation. In light of the patient being lost to follow-up, we acknowledge that a reliable assessment of the effectiveness of the prescribed treatments is not possible.

## 4. Discussion

ARPC is a primary perforating dermatosis, characterized by transepidermal expulsion of altered collagen bundles. The term “acquired” refers to its occurrence in adult patients with coexisting systemic diseases, such as diabetes or renal disease, although other conditions have been postulated in the literature [1,2,3,4,5].

While one case series reported a predominance of the male sex [29] and another one found a majority of female patients [30], the analysis of the rest of the included studies on ARPC identified more women with such disease than men (13 studies vs. 9). On the contrary, regarding the sex distribution in the literature, ARPC has a higher prevalence in males [1].

The mean age of the patients from the included studies was 60.8 years (±14.4) compared to 57 years found in another systematic review [1].

Only one study reported a case of ARPC in the absence of coexisting disease [31], while most of the subjects had comorbidities such as DM (mentioned in 15 studies); cardiovascular diseases (n = 6); malignancies (n = 6) like lung cancer, hepatocellular carcinoma, prostate adenocarcinoma, rectal cancer, thyroid cancer or chronic lymphocytic leukemia; CKD (n = 4); thyroid diseases (n = 3); autoimmune diseases (n = 4); atopic dermatitis and other eczema (n = 4); and infections (n = 4) such as herpes zoster, tinea pedis, HCV infection or HIV infection. Overall, inadequate control of the underlying diseases correlated with the development of ARPC.

Four studies reported cases of drug-induced ARPC, mostly targeted therapies such as erlotinib [46,47] and sorafenib [21,48].

Across all the studies, ARPC clinically presented as multiple papules/nodules with overlying central keratotic, adherent plugs. Whilst the rash was generally symmetric, three studies reported unilateral lesions [18,37,39]. In two case series, the authors reported the lower limbs as the most frequently implied lesion site, followed by the upper limbs and then the trunk [29,30]. A significant correlation between ARPC and the lower extremities was even found [30]. In the rest of the included studies, we found the lesions to appear equally on the lower limbs and the trunk, followed by the upper limbs and rarely occurring on the face. A predilection for extensor surfaces such as the buttocks was observed. Pruritus was present in almost all subjects, with two studies reporting localized itch in diabetic patients [37,39]. In four studies, the lesions elicited the Koebner phenomenon [14,43,46,49].

The major differential diagnoses of ARPC include other perforating dermatoses, as well as prurigo nodularis, multiple keratoacanthomas, lichen planus and arthropod bites [2,3,4,12,13,14,15]. While histopathology remains the gold standard for diagnosing ARPC, additional tools such as dermoscopy or RCM might be helpful, although none exhibits specific features for ARPC, making a differential diagnosis difficult in the absence of a pathology report.

Several studies described the dermoscopic features of ARPC lesions [14,29,49]. The characteristic findings of fully developed lesions included central ulceration/crust, a surrounding white rim and peripheral loop/dotted/branched vessels.

Only one study reported the characteristics of ARPC on reflectance confocal microscopy, such as finding hyper-refractile cord-like structures from dermis to epidermis, corresponding to the extruded collagen fibers [29].

Some studies investigated the pathophysiological mechanisms behind this entity. First, the advanced glycation end products (AGE) and their receptors (RAGE) play a crucial role in this disease. It appears that in ARPC, RAGE is overexpressed in dermal cells like endothelial cells, inflammatory cells and fibroblasts [28], possibly explaining the association between this perforating disorder and conditions that predispose patients to the formation of advanced glycation end products, like aging, DM, CKD, atherosclerosis or various malignancies. RAGE, particularly the CD36 receptor, is implicated in the terminal differentiation of the keratinocytes (KC). Scratching in patients with diabetic or uremic pruritus induces damage to the basement membrane (BM), consequently exposing the basal keratinocytes to AGE such as type I and type III modified collagen. This interaction, mediated via CD36, triggers the terminal differentiation of the KC and their migration upwards on the epidermis along with AGE-modified collagen. Furthermore, this exposure of the KC to altered proteins enhances the expression of the matrix metalloproteinase 9 (MMP-9), which additionally damages the BM, thereby creating a vicious cycle [51].

Second, an imbalance between the matrix metalloproteinases (MMPs) and their tissue inhibitors (TIMPs) degrades the dermal collagen, perpetuating this perforating phenomenon. Type IV collagen appears to be the predominant subtype of the extruded collagen fibers [52].

Third, dysregulation of the TGF-β pathway, particularly with the overexpression of TGF-β3 as a result of coexisting systemic diseases such as DM, contributes to the MMPs/TIMPs imbalance [19].

Furthermore, trauma-induced mechanisms are based on focal necrobiosis, which disrupts the MMPs/TIMPs balance, with the aforementioned consequences.

In addition, Th2-driven inflammation also seems to play a role in the pathogenesis of this entity. The predominance of Th2 cells and their proinflammatory cytokines in the dermis of ARPC patients [27] supports its occurrence in individuals with atopic dermatitis or other eczematous disorders and may explain the efficacy of dupilumab in these cases.

**Figure 3:**
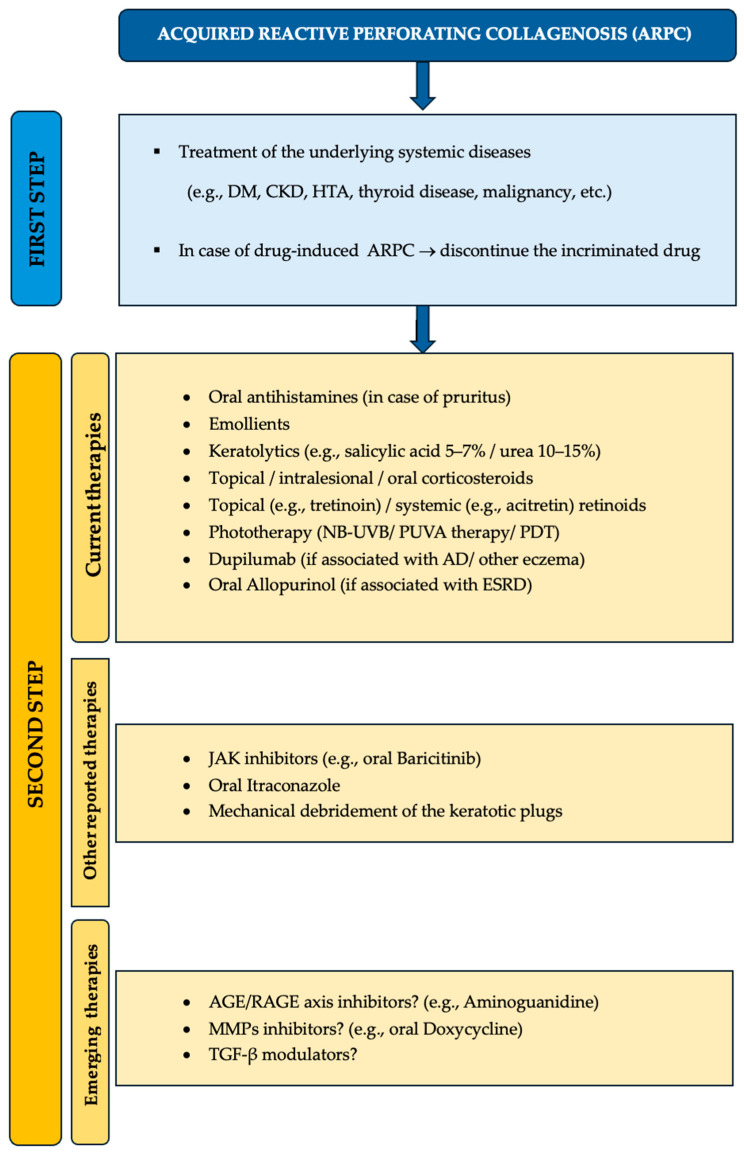
Treatment options for ARPC.

Finally, various therapeutic regimens have been proposed for ARPC (Figure 3), with the treatment being mostly symptomatic. Conventional therapy includes oral antihistamines, topical, intralesional or oral corticosteroids, topical or systemic retinoids, NB-UVB therapy or PUVA therapy. NB-UVB phototherapy and topical corticosteroids modulate pruritus and the inflammatory process, adding substantial benefits to the therapy of ARPC, while retinoids normalize the differentiation of the KC and the MMPs/TIMPs imbalance. Regarding topical corticosteroids, several authors reported clearance of the lesions with alleviation of the pruritus by using a high-potency product, whilst one short communication stated the possibility of developing ARPC following prolonged topical therapy with superpotent dermatocorticoids [50]. An excellent response to dupilumab was observed in several studies [27,29,41,42], especially in patients with a history of AD. JAK inhibitors such as baricitinib were successfully used in a patient with ARPC associated eczema [43]. One study reported the successful treatment of ARPC with oral itraconazole [32].

While oral allopurinol was seen to be more efficient in EPS than in ARPC [30], it could be a preferred therapeutic option in patients with ARPC-associated ESRD [40], as seen in our case report. By inhibiting the xanthine oxidase, allopurinol reduces the production of reactive oxygen species, thereby blocking the formation of AGE with the consequent activation of the KC via the CD36 receptor [40].

Future therapeutic agents might target the AGE–RAGE axis. Aminoguanidine prevents the formation of AGE and could represent a possible candidate for blocking this pathway in ARPC, especially if the perforating process is secondary to diabetes [53].

MMPs inhibitors such as doxycycline represent another therapeutic option, as several studies have demonstrated its efficacy in the treatment of APD [54].

TGF-β modulators might be helpful in ARPC to prevent further disruption of extracellular matrix homeostasis, as observed in this perforating disorder.

## 5. Conclusions

Although uncommon, acquired reactive perforating collagenosis is frequently associated with prevalent systemic disorders such as diabetes mellitus, chronic kidney disease, and cardiovascular conditions. In certain cases, it may even represent the initial or sole clinical manifestation of an underlying disease. Moreover, ARPC has been linked to various malignancies, underscoring the importance of a comprehensive evaluation for systemic disorders and neoplasms once the diagnosis is established. Clinicians should consider ARPC in patients presenting with pruritic papules or nodules exhibiting central keratotic plugs, and histopathological confirmation is essential, typically revealing transepidermal elimination of collagen fibers. Multiple therapeutic approaches have been reported, including topical agents, systemic medications, and phototherapy. However, randomized controlled trials are warranted to establish the most effective management strategies for affected patients.

## Data Availability

The primary data presented in this study are included in Section 3.2. of the main text. No additional datasets were generated.

## Supplementary Materials

The following supporting information can be downloaded at https://www.mdpi.com/article/10.3390/jcm15010391/s1, Table S1: Summary of the included studies. Table S2: PRISMA 2020 checklist. Figure S1: Diagnostic criteria for ARPC.

## References

[1] Karpouzis A., Giatromanolaki A., Sivridis E., Kouskoukis C. (2010). Acquired reactive perforating collagenosis: Current status. J. Dermatol..

[2] Mullins T.B., Sickinger M., Zito P.M. (2024). Reactive Perforating Collagenosis. StatPearls.

[3] Rapini R.P., Bolognia J.L., Schaffer J.V., Cerroni L. (2024). Perforating Diseases. Dermatology.

[4] Wagner G., Sachse M.M. (2013). Acquired reactive perforating dermatosis. JDDG J. Der Dtsch. Dermatol. Ges..

[5] Lynde C.B., Pratt M.D. (2009). Acquired perforating dermatosis: Association with diabetes and renal failure. CMAJ Can. Med. Assoc. J..

[6] Kawakami T., Saito R. (1999). Acquired reactive perforating collagenosis associated with diabetes mellitus: Eight cases that meet Faver’s criteria. Br. J. Dermatol..

[7] Poliak S.C., Lebwohl M.G., Parris A., Prioleau P.G. (1982). Reactive perforating collagenosis associated with diabetes mellitus. N. Engl. J. Med..

[8] Hong S.B., Park J.H., Ihm C.G., Kim N.I. (2004). Acquired perforating dermatosis in patients with chronic renal failure and diabetes mellitus. J. Korean Med. Sci..

[9] Gagnon A.L., Desai T. (2013). Dermatological diseases in patients with chronic kidney disease. J. Nephropathol..

[10] Maurice P.D.L. (1997). Acquired perforating dermatosis in renal patients. Nephrol. Dial. Transplant..

[11] Faver I.R., Daoud M.S., Su W.P.D. (1994). Acquired reactive perforating collagenosis: Report of six cases and review of the literature. J. Am. Acad. Dermatol..

[12] Saray Y., Seçkin D., Bilezikçi B. (2006). Acquired perforating dermatosis: Clinicopathological features in twenty-two cases. J. Eur. Acad. Dermatol. Venereol..

[13] Tsuboi H., Katsuoka K. (2007). Characteristics of acquired reactive perforating collagenosis. J. Dermatol..

[14] Wang C., Liu Y.H., Wang Y.X., Zhang J.Z., Jin J., Guo L.S. (2020). Acquired reactive perforating collagenosis. Chin. Med. J..

[15] Kreuter A., Gambichler T. (2010). Acquired reactive perforating collagenosis. Can. Med. Assoc. J..

[16] Theile-Oche S., Schneider L.A., Reinhold K., Hunzelmann N., Krieg T., Scharffetter-Kochanek K. (2001). Acquired perforating collagenosis: Is it due to damage by scratching?. Br. J. Dermatol..

[17] Bovenmyer D.A. (1970). Reactive perforating collagenosis. Experimental production of the lesion. Arch. Dermatol..

[18] Ghorpade A.K. (2011). Reactive perforating collagenosis with a giant lesion at the site of healed herpes zoster. Indian J. Dermatol. Venereol. Leprol..

[19] Gambichler T., Birkner L., Stücker M., Othlinghaus N., Altmeyer P., Kreuter A. (2009). Up-regulation of transforming growth factor-beta3 and extracellular matrix proteins in acquired reactive perforating collagenosis. J. Am. Acad. Dermatol..

[20] Hinrichs W., Breuckmann F., Altmeyer P., Kreuter A. (2004). Acquired perforating dermatosis: A report on 4 cases associated with scabies infection. J. Am. Acad. Dermatol..

[21] Lederhandler M., Beasley J.M., Brinster N.K., Nagler A.R. (2018). Unusual eruption in association with sorafenib: A case of acquired perforating dermatosis, reactive perforating collagenosis type. Dermatol. Online J..

[22] Yanagihara M., Fujita T., Shirasaki A., Ishiguro K., Kawahara K.I., Ueda K. (1996). The pathogenesis of the transepithelial elimination of the collagen bundles in acquired reactive perforating collagenosis. A light and electron microscopical study. J. Cutan. Pathol..

[23] Saldanha L.F., Gonick H.C., Rodriguez H.J., Marmelzat J.A., Repique E.V., Marcus C.L. (1997). Silicon-related syndrome in dialysis patients. Nephron.

[24] Kawakami T., Soma Y., Mizoguchi M., Saito R. (2001). Immunohistochemical analysis of transforming growth factor-beta3 expression in acquired reactive perforating collagenosis. Br. J. Dermatol..

[25] Liarte S., Bernabé-García Á., Nicolás F.J. (2020). Role of TGF-β in Skin Chronic Wounds: A Keratinocyte Perspective. Cells.

[26] Faler B.J., Macsata R.A., Plummer D., Mishra L., Sidawy A.N. (2006). Transforming growth factor-beta and wound healing. Perspect. Vasc. Surg. Endovasc. Ther..

[27] Liu B., Wu Y., Wu X., Zhong X., Xue R., Zhang Z. (2023). Dupilumab improve acquired reactive perforating collagenosis characterized by type 2 inflammation. Front. Immunol..

[28] Akoglu G., Sungu N., Karaismailoglu E., Aktas A. (2017). Expression of the receptor for advanced glycation end products in acquired reactive perforating collagenosis. Indian J. Dermatol. Venereol. Leprol..

[29] Gao Z., Lu S.J., Shan S.J. (2023). Acquired perforating dermatosis: A clinicopathologic study, and the features of dermoscopy and reflective confocal microscopy of 37 cases. Skin. Res. Technol..

[30] García-Malinis A.J., del Valle Sánchez E., Sánchez-Salas M.P., del Prado E., Coscojuela C., Gilaberte Y. (2017). Acquired perforating dermatosis: Clinicopathological study of 31 cases, emphasizing pathogenesis and treatment. J. Eur. Acad. Dermatol. Venereol..

[31] Gontijo J.R.V., Júnior F.F., Pereira L.B., Pedrosa M.S. (2021). Trauma-induced acquired reactive perforating collagenosis. An. Bras. Dermatol..

[32] Ye B., Cao Y., Liu Y. (2021). Successful treatment of acquired reactive perforating collagenosis with itraconazole. Eur. J. Med. Res..

[33] Ion A., Popa L.G., Porumb-Andrese E., Dorobanțu A.M., Tătar R., Giurcăneanu C., Orzan O.A. (2024). A Current Diagnostic and Therapeutic Challenge: Tinea Capitis. J. Clin. Med..

[34] Ambalathinkal J.J., Phiske M.M., Someshwar S.J. (2022). Acquired reactive perforating collagenosis, a rare entity at uncommon site. Indian J. Pathol. Microbiol..

[35] Fei C., Wang Y., Gong Y., Xu H., Yu Q., Shi Y. (2016). Acquired reactive perforating collagenosis: A report of a typical case. Medicine.

[36] Hasbún C., Sandoval M., González-Bombardiere S. (2020). Case for diagnosis. Hyperpigmented and excoriated papules and nodules in a diabetic patient. An. Bras. Dermatol..

[37] Zhang L.W., Wu J., Xu R.H., Chen T. (2024). Acquired reactive perforating collagenosis in a patient with diabetes. Clevel. Clin. J. Med..

[38] Zhang X., Yang Y., Shao S., Saranathan M. (2020). Acquired reactive perforating collagenosis: A case report and review of the literature. Medicine.

[39] McClure S.P., Liakos W., Seger E.W., Abdo M., Murray A., Gillihan R. (2024). Acquired reactive perforating collagenosis in skin of color. Dermatol. Online J..

[40] Tilz H., Becker J.C., Legat F., Schettini A.P.M., Inzinger M., Massone C. (2013). Allopurinol in the treatment of acquired reactive perforating collagenosis. An. Bras. Dermatol..

[41] Gil-Lianes J., Loughlin C.R.-M., Mascaró J.M. (2022). Reactive perforating collagenosis successfully treated with dupilumab. Australas. J. Dermatol..

[42] Ying Y., Shuang C., Zhen-Ying Z. (2022). Dupilumab may be an alternative option in the treatment of acquired reactive perforating collagenosis combined with AD. Immun. Inflamm. Dis..

[43] Zheng J., Ding Y., Chen Y., Shi Y., Gao Y. (2024). Effectiveness of baricitinib in acquired reactive perforating collagenosis: A case report. Front. Immunol..

[44] Kikuchi N., Ohtsuka M., Yamamoto T. (2013). Acquired reactive perforating collagenosis: A rare association with dermatomyositis. Acta Derm. Venereol..

[45] Huseynova L., Akdogan N., Gököz Ö., Evans S.E. (2020). Acquired reactive perforating collagenosis in association with prostate adenocarcinoma, chronic lymphocytic leukemia, and Graves’ disease. An. Bras. Dermatol..

[46] Jiang X., Song T.T., Hao F. (2021). Erlotinib-induced reactive perforating collagenosis in a case of lung adenocarcinoma. Indian J. Dermatol. Venereol. Leprol..

[47] Suzuki Y., Yamamoto T. (2012). Reactive perforating collagenosis during erlotinib therapy. Acta Derm. Venereol..

[48] Díez D.V., Zubiaur A.G., Montalvo S.M. (2020). Reactive perforating collagenosis: A rare side effect associated with sorafenib. Rev. Esp. Enfermedades Dig..

[49] Su Y., Cui W. (2024). A case report on acquired reactive perforating collagenosis. Medicine.

[50] Madanchi D., Curatolo R., Juratli H.A., Mangas C., Mainetti C. (2024). Ultra-high Potency Topical Corticosteroids as a Potential Trigger for Reactive Perforating Collagenosis. Acta Derm. Venereol..

[51] Fujimoto E., Kobayashi T., Fujimoto N., Akiyama M., Tajima S., Nagai R. (2010). AGE-modified collagens I and III induce keratinocyte terminal differentiation through AGE receptor CD36: Epidermal-dermal interaction in acquired perforating dermatosis. J. Investig. Dermatol..

[52] Herzinger T., Schirren C.G., Sander C.A., Jansen T., Kind P. (1996). Reactive perforating collagenosis--transepidermal elimination of type IV collagen. Clin. Exp. Dermatol..

[53] Thornalley P.J. (2003). Use of aminoguanidine (Pimagedine) to prevent the formation of advanced glycation endproducts. Arch. Biochem. Biophys..

[54] Chai Z. (2019). Acquired perforating dermatosis successfully treated with doxycycline. J. Am. Acad. Dermatol..

